# A secondary analysis of indices of hepatic and beta cell function following 12 weeks of carbohydrate and energy restriction vs. free-living control in adults with type 2 diabetes

**DOI:** 10.1186/s12986-024-00807-x

**Published:** 2024-05-27

**Authors:** Cody Durrer, Hashim Islam, Haoning Howard Cen, Maria Dolores Moya Garzon, Xuchao Lyu, Sean McKelvey, Joel Singer, Alan M. Batterham, Jonathan Z. Long, James D. Johnson, Jonathan P. Little

**Affiliations:** 1 School of Health and Exercise Sciences, University of British Columbia, BC Kelowna, V1V 1V7 Canada; 2 Department of Cellular and Physiological Sciences, Diabetes Research Group, Life Sciences Institute, University of British Columbia, Vancouver, BC Canada; 3 Department of Pathology, Stanford University School of Medicine, Stanford, CA USA; 4 School of Population and Public Health, University of British Columbia, Vancouver, BC Canada; 5 Institute for Personalized Therapeutic Nutrition, Vancouver, BC Canada; 6 Centre for Rehabilitation, School of Health and Life Sciences, Teesside University, Middlesbrough, United Kingdom

## Abstract

**Background:**

Substantial weight loss in people living with type 2 diabetes (T2D) can reduce the need for glucose-lowering medications while concurrently lowering glycemia below the diagnostic threshold for the disease. Furthermore, weight-loss interventions have also been demonstrated to improve aspects of underlying T2D pathophysiology related to ectopic fat in the liver and pancreatic beta-cell function. As such, the purpose of this secondary analysis was to explore the extent to which a low-carbohydrate and energy-restricted (LCER) diet intervention improves markers of beta-cell stress/function, liver fat accumulation, and metabolic related liver function in people with type 2 diabetes.

**Methods:**

We conducted secondary analyses of blood samples from a larger pragmatic community-based parallel-group randomized controlled trial involving a 12-week pharmacist implemented LCER diet (Pharm-TCR: <50 g carbohydrates; ~850–1100 kcal/day; *n* = 20) versus treatment-as-usual (TAU; *n* = 16). Participants were people with T2D, using ≥ 1 glucose-lowering medication, and a body mass index of ≥ 30 kg/m^2^. Main outcomes were C-peptide to proinsulin ratio, circulating microRNA 375 (miR375), homeostatic model assessment (HOMA) beta-cell function (B), fatty liver index (FLI), hepatic steatosis index (HSI), HOMA insulin resistance (IR), and circulating fetuin-A and fibroblast growth factor 21 (FGF21). Data were analysed using linear regression with baseline as a covariate.

**Results:**

There was no observed change in miR375 (*p* = 0.42), C-peptide to proinsulin ratio (*p* = 0.17) or HOMA B (*p* = 0.15). FLI and HSI were reduced by -25.1 (*p* < 0.0001) and − 4.9 (*p* < 0.0001), respectively. HOMA IR was reduced by -46.5% (*p* = 0.011). FGF21 was reduced by -161.2pg/mL (*p* = 0.035) with a similar tendency found for fetuin-A (mean difference: -16.7ng/mL; *p* = 0.11). These improvements in markers of hepatic function were accompanied by reductions in circulating metabolites linked to hepatic insulin resistance (e.g., diacylglycerols, ceramides) in the Pharm TCR group.

**Conclusions:**

The Pharm-TCR intervention did not improve fasting indices of beta-cell stress; however, markers of liver fat accumulation and and liver function were improved, suggesting that a LCER diet can improve some aspects of the underlying pathophysiology of T2D.

**Trial registration:**

Clinicaltrials.gov (NCT03181165).

**Supplementary Information:**

The online version contains supplementary material available at 10.1186/s12986-024-00807-x.

## Introduction

Type 2 diabetes (T2D) – one of the most prevalent lifestyle diseases in the world – puts a massive economic burden on healthcare systems worldwide with an estimated annual cost of ~$760 billion USD [1]. Despite availability of glucose-lowering medications, the prevalence of T2D is on the rise and is projected to continue increasing [1], suggesting that the current glucocentric approach to T2D care is insufficient to stem the rising prevalence of the disease. Nutritional interventions that employ low-carbohydrate [2] or energy restricted [3] strategies leading to weight loss improve glycemia, thereby reducing the need for glucose-lowering medications. There is also evidence that these approaches improve aspects of the underlying T2D pathophysiology (i.e., beta-cell function and insulin sensitivity) [4–7]. Reversal and remission of T2D via nutritional interventions are now recognized as realistic outcomes for managing glycemia in people with T2D [8, 9]; however, for meaningful reversal/remission of the disease to occur, evidence of improvement in the underlying pathophysiology of T2D is needed.

One of the consequences of hyperglycemia and the associated heightened demand for insulin production and secretion in T2D is thought to be the development of endoplasmic reticulum (ER) stress in pancreatic beta-cells [10, 11]. This can be reflected by an increased amount of proinsulin in the circulation for a given amount of C-peptide. In addition to beta-cell related impairments, hepatic steatosis is also highly prevalent among those living with T2D [12]; this is associated with elevated insulin resistance in the liver and aberrant secretion of hepatokines [13, 14]. The presence of hepatic steatosis and T2D is associated with increased secretion of hepatokines that cause inflammation, insulin resistance, and glucose intolerance and decreased secretion/action of hepatokines that are associated with improved insulin sensitivity, improved liver steatosis, and lower adiposity [13, 14]. Low-carbohydrate diets often result in lower postprandial glucose spikes and better glucose variability throughout the day [15, 16]; this relief may allow for beta-cell rest and subsequent alleviation of beta-cell ER stress. When combined with an energy-restricted approach - which rapidly improves liver adiposity, liver insulin sensitivity, and fasting glucose [4] - the resultant reductions in hyperglycemia and liver adiposity could lead to both an alleviation of beta-cell stress and a healthier pattern of hepatokine secretion from the liver.

As such, the purpose of this secondary analysis was to explore the extent to which fasting markers of beta-cell stress/function, liver adiposity, insulin resistance, and hepatokine secretion are improved following the Pharmacist-led Therapeutic Carbohydrate Restriction (Pharm-TCR) intervention.

## Methods

### Study design and participants

Between July 7th 2017 and April 1st 2019 a total of 188 participants were enrolled in a pragmatic community-based randomized controlled trial following a parallel-group design across 12 pharmacies in British Columbia, Canada. Of the 188 participants, 36 are included in the subsample for this secondary exploratory analysis (Pharmacist-led therapeutic carbohydrate- and energy-restricted diet [Pharm-TCR] group: *n* = 20; Treatment-as usual [TAU] control group: *n* = 16). This *n* = 36 subsample includes participants from whom the study team was able to obtain fasting blood samples at baseline and after the 12-week trial; these participants were from five of the twelve original study sites. Fasting blood samples were not able to be obtained from all participants due to pragmatic reasons related to geographical location of the distributed pharmacy sites. The main paper for the study [17] and the trial protocol [18] are published elsewhere and provide details of the primary outcome results, participant recruitment and randomization, and study conduct. Ethical approval for the study was granted by the UBC Clinical Research Ethics Board (H16-01539) and written informed consent was obtained from all participants prior to enrollment in the study. The trial was registered on ClinicalTrials.gov (NCT03181165) on June 8th, 2017.

Participants were eligible for the study if they were able to provide written informed consent, were between the ages of 30–75 years, had been diagnosed with T2D by a physician, were using at least one glucose-lowering medication, and had a body mass index (BMI) of ≥ 30 kg/m^2^. Potential participants were excluded if they had had a heart attack within the previous two years, had any current unstable cardiovascular disorder, had a history of liver disease, kidney disease, or impaired renal function, were currently pregnant, lactating, or planning to become pregnant within the next 12 months, had a diagnosed neurological disorder, a history of bariatric surgery, a history of cancer within the previous five years, or any dietary restrictions that would inhibit adherence to the Pharm-TCR intervention diet.

### Study procedures

Prior to randomization, baseline assessments of height, weight, waist circumference, and blood pressure were performed. Participants then visited a local clinical laboratory where they were assessed for HbA1c, fasting glucose, triglycerides, gamma-glutamyl transferase (GGT), aspartate aminotransferase (AST), and alanine aminotransferase (ALT). The waist circumference, triglycerides, GGT, AST, and ALT results are reported in the main paper [17] but were used in this study to calculate the hepatic steatosis index (HSI) and fatty liver index (FLI). A fasting blood sample was also collected into an EDTA containing vacutainer and processed by the local laboratories. Frozen plasma was sent to our university laboratory and stored at -80 °C for later batch analysis of insulin, proinsulin, C-peptide, microRNA (miR) 375, fetuin-A, and fibroblast growth factor 21 (FGF21).

### Pharm-TCR

Participants in the Pharm-TCR group followed a commercial weight loss plan (Ideal Protein) supplemented with whole foods comprising a daily macronutrient content of < 50 g carbohydrates, ~ 35–45 g fat, and ~ 110–120 g protein for a total of ~ 850–1100 kcal. Participants in this group had weekly visits to the pharmacy that involved meeting with a lifestyle coach and pharmacist to monitor progress and medication usage as well as collect intervention foods. The medication deprescription plan followed by the pharmacists is outlined in the supplementary information of the published protocol paper [18]. On the final visit, height, weight, waist circumference, and blood pressure were assessed and participants were sent to a local clinical laboratory for assessment of the same blood markers that were collected at baseline. A fasting blood sample was again collected, processed using the same procedures at the local laboratories, and frozen plasma sent to our university laboratory for batch analyses with the corresponding baseline samples after storage at -80 °C.

### TAU control

Participants allocated to this group were given standard medication advice by their pharmacist as well as information pamphlets on diet and lifestyle conforming with 2013 Diabetes Canada (formerly the Canadian Diabetes Association) Clinical Practice Guidelines. The TAU group did not attend weekly meetings at the pharmacies during the 12-week period. Upon completion of the 12-weeks, participants returned to the pharmacy and were assessed for height, weight, waist circumference, and blood pressure. Participants were then sent to a local clinical laboratory for assessment of the same blood markers that were collected as baseline following the same procedures as the Pharm-TCR group.

### Outcome measures

#### Insulin, proinsulin and C-peptide

Fasting insulin was assessed in plasma samples with the U-PLEX Human Insulin Assay (intra assay CV: 4.8%; Meso Scale Discovery, Maryland, USA). Fasting proinsulin was measured in plasma samples using via either proinsulin chemiluminescence ELISA (intra assay CV: 3.2%; Alpco, Salem, NH, USA) or the U-PLEX Human Proinsulin Assay (intra assay CV: 2.7%; Meso Scale Discovery, Maryland, USA). Fasting C-peptide was measured in plasma samples via either C-peptide chemiluminescence ELISA (intra assay CV: 7.5%; Alpco, Salem, NH, USA) or the U-PLEX Human C-peptide Assay (intra assay CV: 3.2%; Meso Scale Discovery, Maryland, USA). Baseline and post-study samples from the same participant were assessed using the same assay.

#### Homeostatic model assessment

Insulin resistance was assessed using HOMA IR (HOMA2 Calculator Version 2.2.3). As this is a fasting assessment of insulin resistance, it primarily reflects the liver [19]. Beta-cell function was assessed via the homeostasis model assessment (HOMA) %B (HOMA2 Calculator Version 2.2.3). As recommended by Wallace et al. [20] C-peptide was used in the calculation for HOMA B rather than insulin.

#### Hepatokines and liver adiposity

The hepatokines fetuin-A and FGF21 were assessed in plasma samples collected at baseline and after 12-weeks via ELISA (both Quantikine, R&D Systems Inc, USA). The average intra assay CVs for fetuin-A and FGF21 were 6.3% and 9.7%, respectively. The fasting insulin to C-peptide ratio was calculated as a measure of insulin clearance by the liver. The degree of liver fat accumulation was assessed using the HSI and FLI at baseline and after 12-weeks. Fatty liver index (FLI) = (e ^0.953*log^_e_^(triglycerides) +0.139*BMI+0.718*log^_e_^(GGT) +0.053*waist circumference−15.745^) / (1 + e ^0.953*log^_e_^(triglycerides) +0.139*BMI+0.718*log^_e_^(GGT) +0.053*waist circumference−15.745^) * 100 [21]. Hepatic steatosis index (HSI) = 8 * (ALT/AST) + BMI(+ 2 if T2D, + 2 if female) [22].

#### MicroRNA 375

RNA was extracted from 100 µL plasma using the MagMax *mir*Vana Total RNA Isolation Kit (Applied Biosystems, Foster City, California, USA). 10 ng of total RNA were reverse transcribed into cDNA using TaqMan™ MicroRNA Reverse Transcription Kit (4,366,596, Applied Biosystems). MiR375 levels were determined on the CFX96 Touch Real-Time PCR Detection System (Bio-Rad, Hercules, California, USA) in duplicate qPCR reactions (3.35 ng cDNA/reaction) according to manufacturer’s instructions using the TaqMan™ MicroRNA Assay (#4,427,975, Assay ID 000564, Applied Biosystems) and the TaqMan™ Fast Advanced Master Mix (4,444,556, Applied Biosystems). Synthetic miR375 oligonucleotide (IDT, Iowa, USA) was used to determine cDNA synthesis efficiency and construct standard curves for absolute quantification during qPCR.

#### Anthropometrics and clinical blood markers

Body weight and body fat percentage were assessed using the Tanita model DF-430 U (IL, USA) scale, blood pressure was assessed using the PharmaSmart Model PS-2000 C (BC, Canada), height was measured using the Seca model 700 stadiometer (Germany), and waist circumference was assessed by measuring the distance around the waist at the top of the iliac crest with a tape measure. HbA1c and fasting plasma glucose, along with triglycerides, and liver function tests (GGT, ALT, and AST) were analyzed by provincially-accredited laboratories per standard clinical practice.

#### Untargeted plasma metabolomics

##### Preparation of plasma samples for LC-MS analysis

Polar metabolites were extracted from plasma for LC-MS analysis by adding 150 uL of a 2:1 mixture of acetonitrile/methanol to 50 ul of plasma. This mixture was then centrifuged at 4 °C for 10 min at 15,000 rpm and the supernatant was transferred to a LC-MS vial.

##### Untargeted measurements of metabolites by LC-MS

Untargeted metabolomics measurements were performed on an Agilent 6520 Quadrupole time-of-flight (Q-TOF) LC/MS using electrospray ionization (ESI) in negative mode. The dual ESI source parameters were set as follows, the gas temperature was set at 250 °C with a drying gas flow of 12 L/min and the nebulizer pressure at 20 psi. The capillary voltage was set to 3500 V and the fragmentor voltage set to 100 V. Separation of polar metabolites was conducted using a Luna 5 μm NH2 100 Å LC column (Phenomenex 00B-4378-E0) with normal phase chromatography. Mobile phases were as follows: buffer A, 95:5 water: acetonitrile with 0.2% ammonium hydroxide and 10 mM ammonium acetate; buffer B, acetonitrile. The LC gradient started at 100% B with a flow rate of 0.2 mL min^–1^ from 0 to 2 min. The gradient was then linearly increased to 50% A/50% B at a flow rate of 0.7 mL min^–1^ from 2 to 20 min. From 20 to 25 min, the gradient was maintained at 50% A/50% B at a flow rate of 0.7 mL min^–1^. Differential peak identification was performed with XCMS software.

##### XCMS analysis

LC-MS data were uploaded to Scripps XCMS Online to identify significantly changed metabolites by comparison of the pre (*N* = 19) vs. post samples (*N* = 19). Negative polarity was selected, mass error was set to 20 ppm, mz diff was set to 0.05, minimum peak width was set to 10 and maximum peak width was set to 60. Statistically significant peaks were searched in HMDB (Human Metabolome Database) selecting negative polarity and setting mass error to 20 ppm.

### Statistical analysis

All analyses were performed using R version 4.3.0 [23]. Data were analyzed using linear models with the 12-week outcome value as the dependent variable and fixed effects for treatment (Pharm-TCR vs. TAU), sex, and the baseline outcome values. Model specification was assessed visually using normal probability plots and residuals vs. fitted values plots. When the behaviour of the model residuals warranted a log transformation, effect estimates and 95% confidence intervals were back-transformed to ratio (presented as percentage) differences using the emmeans package version 1.10.1 [24]. Significance was accepted at *p* < 0.05.

For untargeted plasma metabolics analyses, the row z-scores of the altered compounds (*p* < 0.05) detected in untargeted metabolomics were plotted in the heatmap using ComplexHeatmap R package [25]. Because a few outliers (mean +/- 3 SD cutoffs) could drastically skew the z-score, they were excluded from the z-score calculation and are shown in grey color in the heatmap. The log2 fold changes of post- to pre-treatment measurements are calculated for each participant. The median log2 fold changes of each detected compound were plotted in the volcano plot. Pathways from all detected metabolites were predicted using the “Functional Analysis” module in MetaboAnalyst 5.0 online tool [26], following the official tutorial on the website. Briefly, a peak list of all detected compounds is uploaded, containing retention time in minutes and ranked by T scores. Negative ion mode, 20 ppm mass tolerance and “Enforce Primary Ions” are selected in the settings. Pathways are enriched from the human KEGG pathway library, and only pathways with > 3 entries are included. Both Mummichog 2.0 (*p* value cutoff 0.05) and GSEA algorithms (ranked by T scores) are used to predict significant pathways, and their integrated *p* values are plotted. The pathway results are exported from MetaboAnalyst and plotted in R. The R code for metabolomics analysis is available at GitHub repository https://github.com/hcen/pharmTCR_metabolomics.

## Results

### Baseline characteristics of study participants

Thirty-six participants, who had fasting blood samples taken at baseline and following 12-weeks, were included in this analysis. Baseline characteristics are reported in Table 1. Only baseline levels of fetuin-A were different between groups.

**Table 1:** Baseline characteristics

Characteristic	Pharm-TCR (*n* = 20)	TAU (*n* = 16)	*P*-value
Sex			
Male (%)	40.0	37.5	
Female (%)	60.0	62.5	
Body mass (kg)	98.2 ± 18.0	102.7 ± 17.4	0.46
Body mass index (kg/m^2^)	32.4 [30.6–37.6]	32.6 [31.1–37.6]	0.97
Age (years)	58.5 ± 8.3	58.1 ± 12.2	0.90
HbA1c (%)	7.4 ± 0.9	7.9 ± 1.1	0.17
Fasting glucose (mmol/L)	7.9 [6.4–9.3]	8.6 [7.1–10.1]	0.78
Fasting Insulin (pmol/L)	63.4 [30.9-103.5]	110.0 [46.8-124.1]	0.48
Fasting C-peptide (pmol/L)	770.4 ± 412.8	1003.7 ± 446.5	0.12
Fasting proinsulin (pmol/L)	9.5 [6.2–17.9]	7.6 [6.0-16.1]	0.54
Proinsulin to C-peptide ratio	0.01 [0.009–0.02]	0.01 [0.005–0.02]	0.78
Insulin to C-peptide ratio	0.1 [0.06–0.1]	0.09 [0.07–0.1]	0.93
HOMA2 IR	1.3 [0.7–2.3]	2.1 [1.1–2.6]	0.60
HOMA2 B (%)	56.3 ± 28.2	72.4 ± 46.4	0.52
miR375 (starting quantity)	162.2 [116.9-185.9]	129.0 [96.3-193.5]	1.00
Hepatic steatosis index	46.7 [43.3–53.5]	46.4 [44.5–53.5]	0.77
Fatty liver index	84.1 [72.0-95.2]	92.4 [85.5–95.3]	0.26
Fetuin-A (ng/mL)	145.8 [124.1-169.3]	163.9 [137.0-180.5]	0.39
FGF21 (pg/mL)	304.2 [200.8-550.8]	577.0 [478.6-787.5]	0.01
T2D duration (yrs)	11.0 [2.8–16.2]	6.0 [4.0–10.0]	0.60
Medication effect score	1.4 [0.7–2.5]	1.2 [0.7-2.0]	0.24
Diabetes Medications (n %)			
DPP4 Inhibitors	6 (30%)	0 (0%)	
GLP1 Agonists	3 (15%)	4 (25%)	
Insulin	3 (15%)	3 (19%)	
Metformin	19 (95%)	13 (81%)	
SGLT2 Inhibitors	5 (25%)	1 (6%)	
Sulfonylureas	8 (40%)	2 (12%)	
Thiazolidinediones	1 (5%)	0 (0%)	

Data are mean ± standard deviation or median [25th to 75th percentile]. *HbA1c* Hemoglobin A1c, *HOMA2 IR *Homeostatic model assessment insulin resistance, *HOMA2 B *Homeostatic model assessment beta-cell function, *miR375 *Micro RNA 375, *FGF21 *Fibroblast growth factor 21, *GGT *Gamma-glutamyl transferase, *AST *Aspartate transaminase, *ALT *Alanine transaminase, *DPP4 *Dipeptidyl peptidase-4, *GLP1 *Glucagon-like peptide-1, *SGLT2* Sodium-glucose transport protein 2

## Changes following the intervention period

### Body mass and BMI

There was a significant reduction in body mass and BMI (both *p* < 0.001) in the Pharm-TCR group in this subgroup analysis (Table 2). The magnitude of the mean differences is comparable to that in the primary analysis [17]. Changes in diabetes medication use are displayed in Table 3.

**Table 2:** Pharm-TCR treatment effects

Variable	Effect estimate	*p* value
Body mass (kg)	-12.3 (-14.7 to -9.9)	< 0.001
Body mass index (kg/m^2^)	-4.2 (-5.0 to -3.4)	< 0.001
HbA1c (%)	-1.1 (-1.8 to -0.3)	0.0070
Fasting Glucose (mmol/L)	-2.2 (-3.4 to -0.9)	0.0017
Fasting Insulin (pmol/L)	-28.9 (-49.6 to 0.2)% ^a^	0.051
Fasting C-peptide (pmol/L)	-221.5 (-401.0 to -42.0)	0.017
Fasting Proinsulin (pmol/L)	-29.0 (-44.6 to -9.2)% ^a^	0.0080
Proinsulin to C-peptide ratio	-0.002 (-0.004 to 0.0007)	0.17
Insulin to C-peptide ratio	-15.2 (-35.8 to 11.8)% ^a^	0.23
HOMA2 IR	-37.4 (-58.1 to -6.6)% ^a^	0.023
HOMA2 B (%)	10.0 (-3.7 to 23.7)	0.15
miR375 (starting quantity)	-14.2 (-41.5 to 25.7)% ^a^	0.42
Hepatic steatosis index	-4.9 (-6.7 to -3.1)	< 0.001
Fatty liver index	-25.1 (-34.2 to -16.0)	< 0.001
Fetuin-A (ng/mL)	-16.7 (-37.2 to 3.8)	0.11
FGF21 (pg/mL)	-161.2 (-310.0 to -12.5)	0.035

Data are effect estimates (Pharm-TCR treatment effect) and 95% confidence intervals. ^a^Treatment effect and confidence intervals expressed as a percent difference (ratio of geometric means) from log-transformed analysis (Pharm-TCR vs. TAU). *HOMA2 IR *Homeostatic model assessment, *FGF21 *Fibroblast growth factor 21

**Table 3:** Diabetes medication changes

Medication Class	*n*	Deprescribed	Increased	Lowered	No Change
***Pharm-TCR***					
DPP4 Inhibitors	6	6 (100%)	0 (0%)	0 (0%)	0 (0%)
GLP1 Agonists	3	1 (33%)	0 (0%)	0 (0%)	2 (67%)
Insulin	3	1 (33%)	0 (0%)	2 (67%)	0 (0%)
Metformin	19	8 (42%)	0 (0%)	8 (42%)	3 (16%)
SGLT2 Inhibitors	5	4 (80%)	0 (0%)	1 (20%)	0 (0%)
Sulfonylureas	8	8 (100%)	0 (0%)	0 (0%)	0 (0%)
Thiazolidinediones	1	1 (100%)	0 (0%)	0 (0%)	0 (0%)
***TAU***					
DPP4 Inhibitors	0	0 (0%)	0 (0%)	0 (0%)	0 (0%)
GLP1 Agonists	4	0 (0%)	0 (0%)	0 (0%)	4 (100%)
Insulin	3	0 (0%)	0 (0%)	0 (0%)	3 (100%)
Metformin	13	0 (0%)	1 (8%)	0 (0%)	12 (92%)
SGLT2 Inhibitors	1	0 (0%)	0 (0%)	0 (0%)	1 (100%)
Sulfonylureas	2	0 (0%)	1 (50%)	0 (0%)	1 (50%)
Thiazolidinediones	0	0 (0%)	0 (0%)	0 (0%)	0 (0%)

Medication changes are denoted as percentages of participants who were taking each respective medication at baseline. *DPP4 *Dipeptidyl peptidase-4, *GLP1 *Glucagon-like peptide-1, *SGLT2 *Sodium-glucose transport protein 2

### Plasma analytes

There were significant reductions in HbA1c (*p* = 0.007), fasting glucose (*p* = 0.0017), insulin (*p* = 0.045), and proinsulin (*p* = 0.042) following the Pharm-TCR intervention. The hepatokine FGF21 was significantly reduced (*p* = 0.035) in the Pharm-TCR group whereas a similar tendency was found for fetuin-A but this did not reach statistical significance (*p* = 0.11) (Table 2).

### Indices of beta-cell function/stress, liver adiposity and insulin resistance

There was no observed change in the proinsulin to C-peptide ratio (*p* = 0.17), circulating miR375 (*p* = 0.42), or HOMA B (*p* = 0.15; Table 2). There was a significant reduction in both indices of liver fat accumulation, HSI and FLI, in the Pharm-TCR intervention (both *p* < 0.001; Table 2). HOMA2 IR was also significantly reduced following the Pharm-TCR intervention (*p* = 0.023; Table 2). As TZDs and SGLT2 inhibitors have been reported to improve hepatic steatosis, sensitivity analyses excluding the participant taking a TZD at baseline and excluding the six participants taking SGLT2 inhibitors at baseline are included in Table S1 and Table S2, respectively (Additional file 1). Furthermore, although HOMA2 IR can be calculated in patients taking exogenous insulin provided blood samples are taken when glucose and insulin concentrations are at a steady-state prior to medication use [20], and HOMA2 B can be calculated in these instances using C-peptide rather than insulin [20], an analysis of the data excluding participants taking exogenous insulin is provided in Table S3 (Additional file 1). In both of these sensitivity analyses, the findings were unchanged.

### Plasma metabolites

**Fig. 1:**
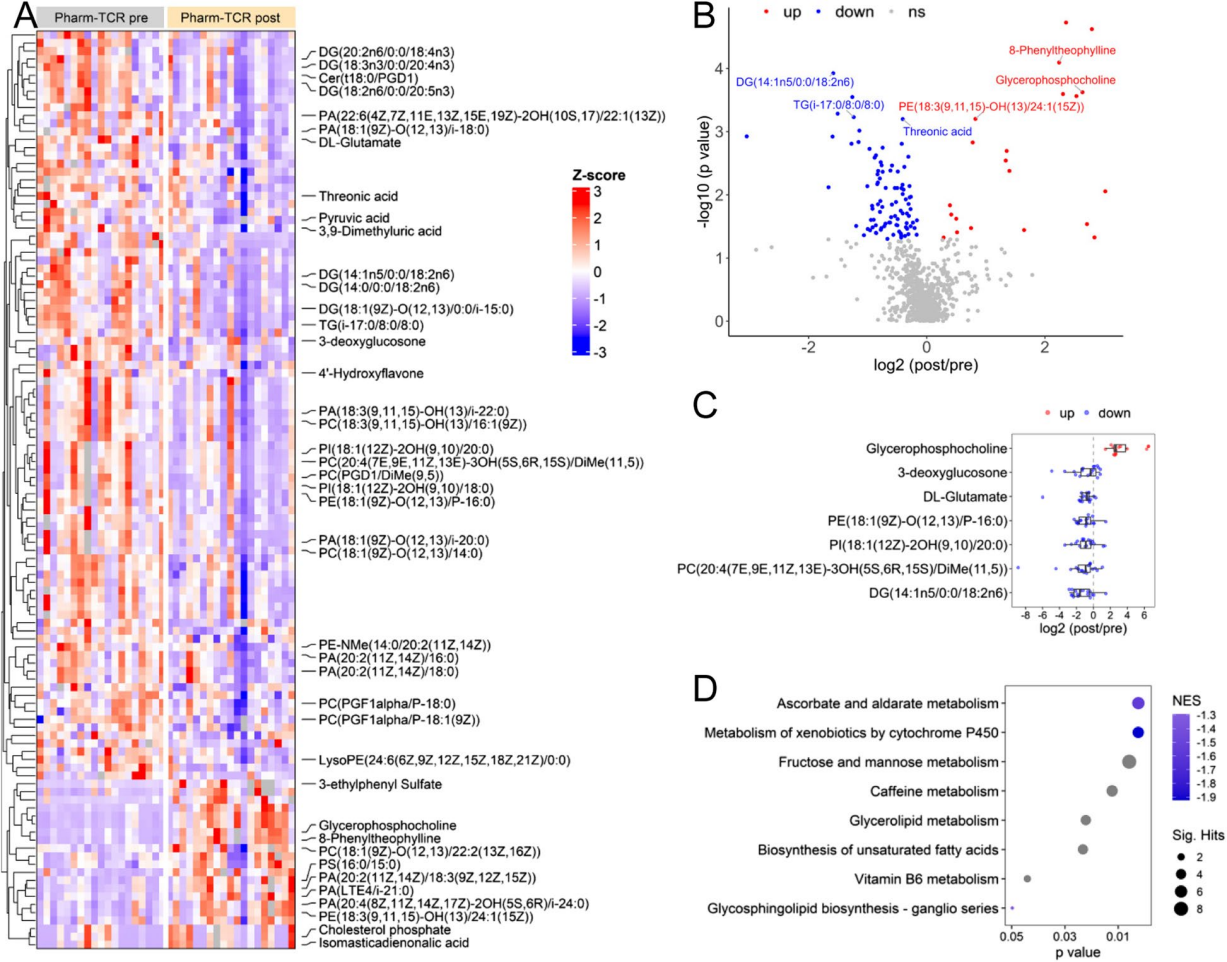
Untargeted metabolomics of the plasma sample at post vs. pre pharm-TCR treatment. **A** The heatmap of all the altered metabolites (*p* < 0.05), among which the identified metabolites are labelled. **B** The volcano plot showing the log2 fold change (post/pre treatment) and –log10 (p value) of all detected metabolites. The metabolites with the most significant changes (FDR < 0.05) are labeled. **C** The log2 fold change (post/pre treatment) of selected metabolites. Each dot represents one participant. **D** Predicted pathways enriched using integrated Mummichog and GSEA methods. From GSEA, two pathways showed negative NES (normalized enrichment score), suggesting the metabolites in these pathways are mostly downregulated, while other pathways had undetermined directions

A total of 114 metabolites were altered from pre- to post-intervention in the Pharm-TCR intervention with 21 metabolites upregulated and 93 metabolites downregulated (Fig. 1A, *p* < 0.05). The 42 metabolites identified based on our mass error threshold of 20 ppm are labelled in Fig. 1A. The most significantly altered metabolites are shown in Fig. 1B. Notable downregulated metabolites linked to T2D pathophysiology included diacylglycerol, triacylglycerol, ceramide, phosphatidylcholine, phosphatidylethanolamine, and phosphatidylinositol species (Fig. 1A, C). Two pathways (ascorbate and alderate metabolism, metabolism of xenobiotics by cytochrome p50) showed negative normalized enrichment score (NES) while other pathways had undetermined directions (Fig. 1D).

## Discussion

The present secondary analysis of a subgroup of participants in the Pharm-TCR trial provides evidence that a LCER diet in people living with type 2 diabetes may also improve some of the underlying pathophysiological defects in T2D. Namely, we observed improvements in markers of liver fat accumulation, insulin sensitivity, and hepatokine secretion.

The ratio of proinsulin to C-peptide is considered a marker of beta-cell ER stress, as the relative amount of improperly processed proinsulin released into the circulation increases as ER stress increases [10, 11]. C-peptide, rather than insulin, is preferable to compare to proinsulin as this method removes any influence of insulin clearance by the liver. In this study, we did not observe a reduction in the proinsulin to C-peptide ratio despite the fact that glycemia (i.e., HbA1c and fasting glucose) insulin, proinsulin, and C-peptide were reduced in the Pharm-TCR group; however, it is possible that a more dynamic assessment of beta-cell function than what can be observed from fasting blood samples (e.g., oral glucose tolerance test) is required to observe a reduction in the proinsulin to C-peptide ratio. In support of this, a recent study by Mezza et al., assessed the similar proinsulin to insulin ratio as a measure of beta-cell stress during a mixed-meal tolerance test before and after a 50% pancreatectomy [27]. Although the loss of 50% of one’s beta-cells should be enough to induce stress during a meal challenge in the remaining beta-cells, they only observed a significant increase in the proinsulin to insulin ratio 180 min into the test [27]. In contrast, Skytte et al. observed an improvement in fasting proinsulin to insulin ratio after six weeks of dietary carbohydrate restriction [7]; however, people with T2D who were taking injectable diabetes medications (e.g., insulin) were excluded from that study, which likely led to a more recently diagnosed and less severe T2D disease status.

In addition to the absence of an improvement in the proinsulin to C-peptide ratio, we also did not observe an improvement in circulating levels of miR375 – a proposed marker of beta-cell stress that has been described in both type 1 diabetes (T1D) and T2D [28, 29]. MicroRNA 375 is typically elevated in recent onset T1D, as well as in people with prediabetes and T2D (reviewed in 19). It is possible that absence of an effect on miR375 could be due to the fact that the rate of beta-cell death was not high enough to be impacted (i.e., a floor effect); however, it is also possible that the intervention simply did not impact beta-cell death. Finally, we also did not observe a change in HOMA2 B, but the fasting nature of the outcome is a limitation and a dynamic assessment of beta-cell function would have provided a broader perspective. Taken together, we did not observe any evidence of improvements in beta-cell function or stress outcomes. The advanced duration of T2D in the Pharm TCR group and use of sulfonylureas – both of which may adversely impact the capacity of beta cells to recover [5, 30] – may have contributed to the lack of improvements in indices of beta cell function in our intervention.

As expected with any intervention that induces substantial weight loss, we observed improvements in both fatty liver disease indices (i.e., HSI and FLI) following the Pharm-TCR intervention. We also observed a significant reduction in the hepatokine FGF21 and a potential reduction in fetuin-A. Although considered a “good” hepatokine due to favourable effects on insulin sensitivity [31], FGF21 is chronically elevated in T2D [32] and the secretion of this hepatokine can be regulated by the circulating glucagon to insulin ratio [33]. As such, insulin resistance by the liver is speculated to play a role in mediating the higher circulating levels of FGF21 seen in T2D [33]. Fetuin-A is also increased in circulation in T2D and, unlike FGF21, causes insulin resistance and proinflammatory cytokine production from adipocytes and macrophages [34]. As such, lower circulating levels of these hepatokines following the Pharm-TCR intervention indicates a transition towards a healthy hepatokine secretion pattern. In addition to improvements in indices of liver fat accumulation and hepatokine secretion, the Pharm-TCR intervention also reduced insulin resistance as assessed by HOMA2 IR. Since HOMA is based off fasting measures of insulin and blood glucose, it primarily reflects liver insulin resistance [19]. A reduction in hepatokine secretion combined with improvements in HSI, FLI, and HOMA2 IR indicate that the Pharm-TCR intervention likely reduced liver fat accumulation and improved metabolic related liver function. This suggests that the intervention had a positive impact on liver pathophysiology in addition to improving measures of glycemia.

To further investigate the impact of the Pharm-TCR intervention on biomarkers linked to T2D pathophysiology, we performed exploratory untargeted metabolics analysis of plasma samples from the treatment group. We found significant reductions in various glycerolipid (di- and triacylglycerol) and ceramide species known to contribute to hepatic insulin resistance [35], thereby supporting the observed improvements in markers of liver fat and metabolic function in the Pharm TCR group. Moreover, several metabolites recently identified as markers of T2D risk (e.g., phosphatidylcholines, phosphatidylethanolamines, phosphatidylinositols) [36] were lowered after the intervention in the Pharm TCR group. Althought most of the metabolites identified were downregulated, glycerophosphocoline – an inverse independent predictor of T2D risk that is reduced in T2D [37] - was among the most robustly increased metabolites following the intervention. Collectively, these data support an improvement in certain aspects of the underlying T2D pathophysiology in the Pharm TCR group.

While the results from this study are positive, there are several limitations that must be addressed in this exploratory secondary analysis. First, although the main trial was a randomized controlled trial, the participants included in this secondary analysis are based off availability of fasting blood samples at baseline and following the 12-week intervention. Although there were no baseline imbalances in most outcomes (with the exception of FGF21), the use of a subsample allows the possibility that the randomization has been broken. In line with this, while there was no difference in baseline medication effect score (an index of the overall intensity of a pharmacological glucose-lowering regimen), there appears to be group imbalances in the use of individual medications in this subgroup. Regardless, mean reductions in HbA1c and body mass were consistent with those in the main paper, suggesting a representative subsample in those outcomes. In any case, the results of this secondary analysis need to be interpreted with caution. We chose not to adjust the criterion p-values for multiple testing (apart from the metabolomics analysis) given the exploratory nature of this study. The indirect nature of the outcome assessments in this study represents another limitation. Although it was not feasible, nor the purpose, of the main trial to assess these outcomes, the consistency in the results of this study (in particular, those regarding the liver) give us confidence that the findings are robust. It is important to note that the results of this study occurred with simultaneous reductions in diabetes medication use and the findings should be interpreted as such. Finally, the beta-cell function markers in this study were limited to fasting blood samples and therefore may not reflect the dynamic responses seen during a glucose challenge or meal.

In conclusion, the Pharm-TCR intervention resulted in no change in beta-cell outcomes, but did improve indices of liver fat accumulation, hepatokine secretion, and insulin sensitivity. Taken together, these findings suggest that a low-carbohydrate energy restricted diet represents a viable strategy for not only reducing hyperglycemia but also improving some of the underlying pathophysiological drivers of T2D in individuals with the disease.

### Supplementary Information

Supplementary Material 1.

## Data Availability

Data are available upon resonable request made to the corresponding author (JPL).
