# Supplementary material for: A secondary analysis of indices of hepatic and beta cell function following 12 weeks of carbohydrate and energy restriction vs. free-living control in adults with type 2 diabetes
Source: Nutr Metab (Lond). 2024 May 27;21:29. doi: 10.1186/s12986-024-00807-x (PMC11129411; doi:10.1186/s12986-024-00807-x)
Supplement: Supplementary file 1 — Supplementary Material 1. [file 12986_2024_807_MOESM1_ESM.docx]

| **Supplementary Table 1.** Pharm-TCR treatment effects | | |
| --- | --- | --- |
| Variable | Effect estimate | p value |
| Body mass (kg) | -11.8 (-14.1 to -9.5) | <0.001 |
| Body mass index (kg/m^2^) | -4.1 (-4.8 to -3.3) | <0.001 |
| HbA1c (%) | -1.0 (-1.8 to -0.2) | 0.014 |
| Fasting Glucose (mmol/L) | -2.2 (-3.5 to -0.9) | 0.0015 |
| Fasting Insulin (pmol/L) | -29.8 (-50.5 to -0.3)% * | 0.048 |
| Fasting C-peptide (pmol/L) | -237.8 (-418.5 to -57.0) | 0.012 |
| Fasting Proinsulin (pmol/L) | -30.1 (-45.6 to -10.0)% * | 0.0069 |
| Proinsulin to C-peptide ratio | -0.002 (-0.004 to 0.0009) | 0.20 |
| Insulin to C-peptide ratio | -15.4 (-36.4 to 12.5)% * | 0.24 |
| HOMA2 IR | -38.3 (-59.0 to -6.9)% * | 0.023 |
| HOMA2 B (%) | 9.9 (-4.4 to 24.1) | 0.17 |
| miR375 (starting quantity) | -12.6 (-40.8 to 29.0)% * | 0.48 |
| Hepatic steatosis index | -4.9 (-6.8 to -3.1) | <0.001 |
| Fatty liver index | -26.5 (-35.7 to -17.2) | <0.001 |
| Fetuin-A (ng/mL) | -15.5 (-36.4 to 5.5) | 0.14 |
| FGF21 (pg/mL) | -160.6 (-313.2 to -8.0) | 0.040 |
| Data are effect estimates (Pharm-TCR treatment effect) and 95% confidence intervals from the analysis excluding participants taking thiazolidinediones (n=1). *Treatment effect and confidence intervals expressed as a percent difference (ratio of geometric means) from log-transformed analysis (Pharm-TCR vs. TAU). HOMA2 IR: homeostatic model assessment; FGF21: fibroblast growth factor 21. | | |

| **Supplementary Table 2.** Pharm-TCR treatment effects | | |
| --- | --- | --- |
| Variable | Effect estimate | p value |
| Body mass (kg) | -11.3 (-13.5 to -9.1) | <0.001 |
| Body mass index (kg/m^2^) | -3.9 (-4.6 to -3.2) | <0.001 |
| HbA1c (%) | -1.1 (-2.0 to -0.2) | 0.021 |
| Fasting Glucose (mmol/L) | -2.3 (-3.7 to -0.8) | 0.0034 |
| Fasting Insulin (pmol/L) | -38.5 (-54.2 to -17.3)% * | 0.0023 |
| Fasting C-peptide (pmol/L) | -307.6 (-477.1 to -138.1) | <0.001 |
| Fasting Proinsulin (pmol/L) | -33.1 (-49.4 to -11.4)% * | 0.0068 |
| Proinsulin to C-peptide ratio | -0.0008 (-0.003 to 0.002) | 0.52 |
| Insulin to C-peptide ratio | -13.2 (-32.3 to 11.2)% * | 0.25 |
| HOMA2 IR | -48.5 (-63.4 to -27.6)% * | <0.001 |
| HOMA2 B (%) | 5.0 (-10.4 to 20.5) | 0.51 |
| miR375 (starting quantity) | -6.1 (-38.3 to 43.0)% * | 0.76 |
| Hepatic steatosis index | -4.9 (-6.9 to -2.8) | <0.001 |
| Fatty liver index | -25.9 (-36.3 to -15.5) | <0.001 |
| Fetuin-A (ng/mL) | -9.4 (-30.5 to 11.6) | 0.37 |
| FGF21 (pg/mL) | -111.6 (-268.2 to 45.0) | 0.16 |
| Data are effect estimates (Pharm-TCR treatment effect) and 95% confidence intervals from the analysis excluding participants taking SGLT2 inhibitors (n=6). *Treatment effect and confidence intervals expressed as a percent difference (ratio of geometric means) from log-transformed analysis (Pharm-TCR vs. TAU). HOMA2 IR: homeostatic model assessment; FGF21: fibroblast growth factor 21. | | |

| **Supplementary Table 3.** Pharm-TCR treatment effects | | |
| --- | --- | --- |
| Variable | Effect estimate | p value |
| Body mass (kg) | -12.1 (-14.5 to -9.6) | <0.001 |
| Body mass index (kg/m^2^) | -4.0 (-4.8 to -3.1) | <0.001 |
| HbA1c (%) | -1.1 (-2.0 to -0.3) | 0.011 |
| Fasting Glucose (mmol/L) | -2.2 (-3.6 to -0.7) | 0.0063 |
| Fasting Insulin (pmol/L) | -29.3 (-50.9 to 1.9)% * | 0.062 |
| Fasting C-peptide (pmol/L) | -242.5 (-433.5 to -51.5) | 0.015 |
| Fasting Proinsulin (pmol/L) | -35.3 (-50.3 to -15.9)% * | 0.0021 |
| Proinsulin to C-peptide ratio | -0.002 (-0.005 to 0.0008) | 0.17 |
| Insulin to C-peptide ratio | -12.6 (-27.8 to 5.8)% * | 0.16 |
| HOMA2 IR | -41.4 (-62.4 to -8.6)% * | 0.021 |
| HOMA2 B (%) | 4.6 (-11.1 to 20.3) | 0.55 |
| miR375 (starting quantity) | -1.4 (-35.0 to 49.5)% * | 0.94 |
| Hepatic steatosis index | -5.5 (-7.5 to -3.6) | <0.001 |
| Fatty liver index | -26.8 (-37.2 to -16.4) | <0.001 |
| Fetuin-A (ng/mL) | -13.0 (-34.1 to 8.1) | 0.22 |
| FGF21 (pg/mL) | -183.8 (-350.3 to -17.3) | 0.032 |
| Data are effect estimates (Pharm-TCR treatment effect) and 95% confidence intervals from the analysis excluding participants taking exogenous insulin (n=6). *Treatment effect and confidence intervals expressed as a percent difference (ratio of geometric means) from log-transformed analysis (Pharm-TCR vs. TAU). HOMA2 IR: homeostatic model assessment; FGF21: fibroblast growth factor 21. | | |

| **Supplementary Table 4.** Pharm-TCR treatment effects disaggregated by sex | | |
| --- | --- | --- |
| Variable | Effect | Effect estimate |
| Body mass (kg) | Male | -12.5 (-16.4 to -8.6) |
|  | Female | -12.1 (-15.2 to -9.0) |
|  | Difference | -0.4 (-5.3 to 4.6) |
| Body mass index (kg/m^2^) | Male | -3.8 (-5.0 to -2.5) |
|  | Female | -4.5 (-5.5 to -3.5) |
|  | Difference | 0.7 (-0.9 to 2.3) |
| HbA1c (%) | Male | -1.1 (-2.3 to 0.1) |
|  | Female | -1.1 (-2.1 to -0.02) |
|  | Difference | -0.04 (-1.7 to 1.6) |
| Fasting Glucose (mmol/L) | Male | -0.7 (-2.5 to 1.2) |
|  | Female | -3.2 (-4.8 to -1.6) |
|  | Difference | 2.5 (0.1 to 5.0) |
| Fasting Insulin (pmol/L) | Male | -33.1 (-61.6 to 16.4)% * |
|  | Female | -26.1 (-52.8 to 15.7)% * |
|  | Difference | -9.5 (-55.5 to 84.1)% * |
| Fasting C-peptide (pmol/L) | Male | -124.0 (-415.2 to 167.3) |
|  | Female | -277.9 (-501.5 to -54.2) |
|  | Difference | 153.9 (-206.8 to 514.6) |
| Fasting Proinsulin (pmol/L) | Male | -26.9 (-51.2 to 9.5)% * |
|  | Female | -30.4 (-49.5 to -4.0)% * |
|  | Difference | 5.0 (-37.3 to 75.9)% * |
| Proinsulin to C-peptide ratio | Male | -0.002 (-0.006 to 0.002) |
|  | Female | -0.001 (-0.004 to 0.002) |
|  | Difference | -0.0008 (-0.006 to 0.004) |
| Insulin to C-peptide ratio | Male | -28.5 (-54.5 to 12.3)% * |
|  | Female | -5.6 (-34.0 to 34.9)% * |
|  | Difference | -24.3 (-57.7 to 35.4)% * |
| HOMA2 IR | Male | -31.8 (-63.2 to 26.2)% * |
|  | Female | -41.4 (-65.7 to 0.3)% * |
|  | Difference | 16.2 (-48.3 to 161.3)% * |
| HOMA2 B (%) | Male | -0.7 (-22.6 to 21.1) |
|  | Female | 16.6 (-0.6 to 33.7) |
|  | Difference | -17.3 (-44.9 to 10.4) |
| miR375 (starting quantity) | Male | -26.5 (-61.9 to 41.9)% * |
|  | Female | -6.5 (-42.7 to 52.6)% * |
|  | Difference | -21.4 (-65.8 to 80.5)% * |
| Hepatic steatosis index | Male | -5.8 (-8.7 to -3.0) |
|  | Female | -4.3 (-6.6 to -2.1) |
|  | Difference | -1.5 (-5.2 to 2.2) |
| Fatty liver index | Male | -33.5 (-46.0 to -21.0) |
|  | Female | -17.4 (-29.3 to -5.5) |
|  | Difference | -16.1 (-33.2 to 1.0) |
| Fetuin-A (ng/mL) | Male | -26.2 (-59.1 to 6.7) |
|  | Female | -10.6 (-37.1 to 15.9) |
|  | Difference | -15.6 (-57.8 to 26.5) |
| FGF21 (pg/mL) | Male | 70.1 (-136.9 to 277.1) |
|  | Female | -305.9 (-472.0 to -139.8) |
|  | Difference | 376.0 (118.8 to 633.3) |
| Data are effect estimates (Pharm-TCR treatment effect) and 95% confidence Intervals from the analysis excluding participants taking exogenous insulin (n=6). *Treatment effect and confidence Intervals expressed as a percent difference (ratio of geometric means) from log-transformed analysis (Pharm-TCR vs. TAU). HOMA2 IR: homeostatic model assessment; FGF21: fibroblast growth factor 21. | | |
